# Dissection of α_4_β_7_ integrin regulation by Rap1 using novel conformation-specific monoclonal anti-β_7_ antibodies

**DOI:** 10.1038/s41598-020-70111-0

**Published:** 2020-08-06

**Authors:** Tsuyoshi Sato, Sayaka Ishihara, Ryoya Marui, Junichi Takagi, Koko Katagiri

**Affiliations:** 1 Department of Biosciences, School of Science, Kitasato University, 1-15-1 Kitasato, Minamiku, Sagamihara, Kanagawa 252-0373 Japan; 2 Laboratory of Protein Synthesis and Expression, Institute for Protein Research, Osaka University, Osaka, Japan

**Keywords:** Cell biology, Immunology

## Abstract

Integrin activation is associated with conformational regulation. In this study, we developed a system to evaluate conformational changes in α_4_β_7_ integrin. We first inserted the PA tag into the plexin-semaphorin-integrin (PSI) domain of β_7_ chain, which reacted with an anti-PA tag antibody (NZ-1) in an Mn^2+^-dependent manner. The small GTPase Rap1 deficiency, as well as chemokine stimulation and the introduction of the active form of Rap1, Rap1V12, enhanced the binding of NZ-1 to the PA-tagged mutant integrin, and increased the binding affinity to mucosal addressing cell adhesion molecule-1 (MAdCAM-1). Furthermore, we generated two kinds of hybridomas producing monoclonal antibodies (mAbs) that recognized Mn^2+^-dependent epitopes of β_7_. Both epitopes were exposed to bind to mAbs on the cells by the introduction of Rap1V12. Although one epitope in the PSI domain of β_7_ was exposed on Rap1-deficienct cells, the other epitope in the hybrid domain of β_7_ was not. These data indicate that the conversion of Rap1-GDP to GTP exerts two distinct effects stepwise on the conformation of α_4_β_7_. The induction of colitis by Rap1-deficient CD4^+^ effector/memory T cells suggests that the removal of constraining effect by Rap1-GDP on α_4_β_7_ is sufficient for homing of these pathogenic T cells into colon lamina propria (LP).

## Introduction

Lymphocyte adhesion and migration are important for the generation and execution of immune and inflammatory responses. Integrins are a family of α/β heterodimeric adhesion receptors that transmit signals bi-directionally across the plasma membrane^1–3^. In the multistep leukocyte adhesion cascade, selectins generally mediate rolling, and integrins mediate subsequent arrest. In contrast, the gut homing integrin α_4_β_7_ mediates leukocyte rolling and arrest in vivo^4^. MAdCAM-1, a ligand for α_4_β_7_, is constitutively expressed in postcapillary venules of intestinal lamina propria (LP) and acts as a key addressin for intestinal homing^5^. Therefore, the adhesive activity of α_4_β_7_ directly reflects the ability of cells to move to the mucosal tissues of the intestine.

Regulation of T-cell trafficking by both Rap1-GTP and -GDP is a key control mechanism of the lymphocyte adhesion cascade^6^. Rap1-GTP recruits downstream effectors, such as RAPL (regulator of cell polarization and adhesion enriched in lymphoid tissues), which binds integrin α chain, and RIAM (RAP1-interacting adapter molecule) and talin which bind integrin β chain^7–9^. Rap1-GDP suppresses lymphocyte rolling behaviors via activation of LOK (lymphocyte-orientated kinase) and phosphorylation of ERM (ezrin, radixin and moesin)^10^.

The T cell number in mesenteric lymph nodes is important for mucosal tolerance. Integrin activation by Rap1-GTP plays an important role in the circulation of naive T (T_N_) cells, whereas Rap1-GDP in resting T_N_ and effector/memory T (T_EM_) cells limits rolling behaviors in blood vessels and retards lymphocyte homing^10^. Therefore, Rap1 deficiency leads to lymphopenia and the generation of pathogenic T_EM_ cells in lymph nodes. Furthermore, it facilitates homing of T_EM_ cells into the colon, which exacerbates spontaneous T-cell-dependent colitis and tubular adenomas^10^. Excess infiltration of T_EM_ cells by Rap1 deficiency points to the involvement of Rap1-GDP in the regulation of the activation of α_4_β_7_.

Binding of α_4_β_7_ to MAdCAM-1 with high affinity is critical step for lymphocyte arrest. The regulation of the ligand-binding affinity is associated with conformational rearrangement of the integrin molecule^11^. Previous studies showed that integrin extracellular domains existed in distinct global conformational states that differed in their affinity for ligands: a low-affinity bent conformation with a close headpiece and a high-affinity extended conformation with an open head piece^12–14^. The equilibrium among these different states is regulated by integrin inside-out signaling and extracellular stimuli, such as divalent cations. Compared with the low-affinity state in Ca^2+^/Mg^2+^, the removal of Ca^2+^ or the addition of Mn^2+^ results in a marked increase in ligand-binding of almost all integrins^15^. There are few conventional activation-specific antibodies to recognize only activated α_4_β_7_^16,17^.

In this study, to explore the effects of Rap1 deficiency on the conformation of α_4_β_7_, we developed an epitope grafting system to detect conformational changes in α_4_β_7_ by inserting a PA tag into the PSI domain of the β_7_ chain. The PA tag was a 12-residue peptide (GVAMPGARDDVV) derived from human podoplanin, which is recognized by NZ-1^18^. We also prepared one rat mAb (G3 mAb), in which the epitope was located in the PSI domain, as was the case with the grafting site of the PA tag. Using NZ-1 or G3 mAb, we revealed that Rap1 deficiency, as well as chemokine stimulation, induced the active conformation of α_4_β_7_, suggesting that Rap1-GDP restricts conformation of α_4_β_7_ in an inactive state. The other rat mAb (H3 mAb), in which the epitope was located in the hybrid domain of the β_7_ chain, recognized the active conformation of α_4_β_7_ on Rap1V12-expressing cells, but not that on Rap1-deficient cells. These data suggested that Rap1-GTP further promoted conformational change leading to high-affinity α_4_β_7_. Consistent with these data, Rap1-deficient CD4^+^ T_EM_ cells (pathogenic T cells), which home to colon LP in a α_4_β_7_-MAdCAM-1-dependent manner and induce colitis, exhibited increased expression of the epitope recognized by G3 mAb but not H3 mAb. Thus, constraining effect by Rap1-GDP on α_4_β_7_ presumably contributes to suppress excess infiltration of pathogenic T cells into colon LP and prevent the development of colitis.

## Results

### Development of a system to measure the active conformation of α_4_β_7_ by inserting a PA tag

**Figure 1:**
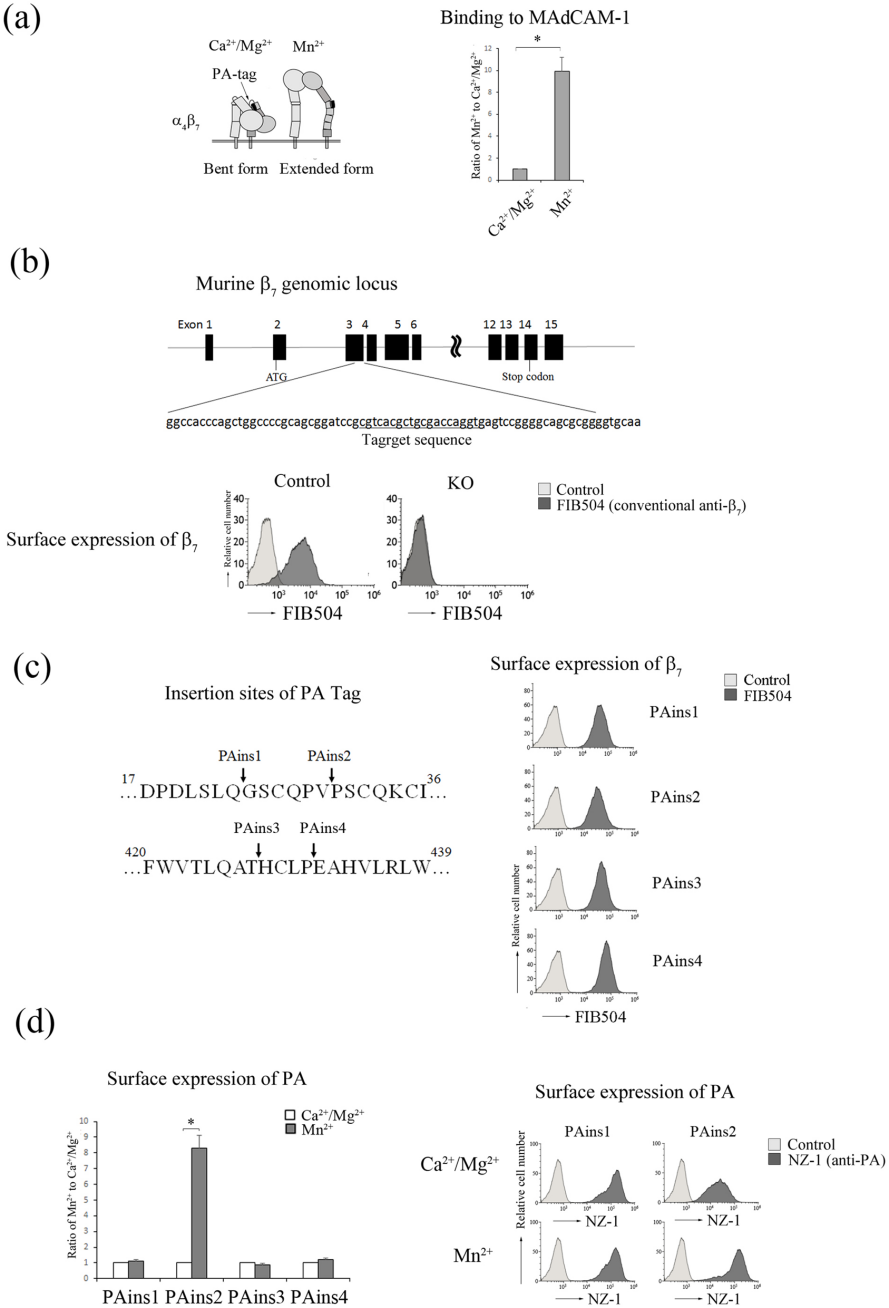
Detection of conformational changes in α_4_β_7_ using a PA tag. (**a**) (Left) A model of the conformational states of the extracellular domain of α_4_β_7_: bent form with a closed headpiece in low affinity and extended form with an open headpiece in high affinity. (Right) The binding of wild-type (wt) BAF cells to MAdCAM-1 in the presence of 1 mM Ca^2+^/Mg^2+^, or 0.5 mM Mn^2+^. The IMF (Mean Fluorescence Intensity) of binding to MAdCAM-1-Fc was normalized to the IMF of anti-β_7_ (FIB504). The IMF is presented as the fold-increase relative to that of wt cells in the presence of 1 mM Ca^2+^/Mg^2+^ values of 1. Data represent the mean ± SE of three independent experiments. *P < 0.005, versus in the presence of Ca^2+^/Mg^2+^. (**b**) (Upper) Systematic overview of the gene targeting strategy. The position of the single-guide RNA target site of exon 3 is indicated by an underline. (Lower) Flow cytometry profiles of anti-β_7_ of control or knockout cells using CRISPR/Cas9-mediated genome editing is shown. (**c**) (Left) Insertion sites of the PA tag. The PA tag sequence was inserted at the indicated sites of the β_7_ sequence in the designed mutants (PAins 1–4). (Right) Flow cytometry profiles of anti-β_7_ of BAF cells transfected with each designed mutant. (**d**) (Left) The binding of NZ-1 to each transfectant in the presence of 1 mM Ca^2+^/Mg^2+^, or 0.5 mM Mn^2+^. The IMF of binding of each transfectant to NZ-1 was normalized to the IMF of anti-β_7_ and is presented as the fold-increase relative to that of each transfectant in the presence of 1 mM Ca^2+^/Mg^2+^ values of 1. Data represent the mean ± SE of three independent experiments. (Right) Flow cytometry profiles of NZ-1 of PAins1 and PAins2 in the presence of 1 mM Ca^2+^/Mg^2+^, or 0.5 mM Mn^2+^. *P < 0.001, versus in the presence of Ca^2+^/Mg^2+^.

α_4_β_7_ showed low-affinity state to MAdCAM-1 in Ca^2+^/Mg^2+^, and the addition of Mn^2+^ increased the binding affinity of α_4_β_7_ to MAdCAM-1 (Fig. 1a). To probe the conformational state of α_4_β_7_ using the PA-tag-NZ-1 system (Fig. 1a), a proB cell line (BAF cells), in which the β_7_ chain was knocked-out using CRISPR/Cas9-mediated genome editing, and β_7_ chains cDNA which were inserted PA tag into 4 locations (PAins 1: 23/24, PAins 2: 29/30, PAins 3: 427/428, PAins 4: 431/432) were introduced into BAF cells (Fig. 1b,c). These insertion mutants of β_7_-expressing cells were stained with NZ-1 or FIB504 (conventional mAb against mouse/human β_7_, which recognizes the binding sites of α_4_β_7_ with MAdCAM-1) in the presence of 1 mM Ca^2+^/Mg^2+^ or 0.5 mM Mn^2+^, and analyzed by flow cytometry. All mutants of β_7_ were approximately equally expressed on the cell surface (Fig. 1c). PA expression on the surface of PAins2-expressing cells (PAins2 cells) was reduced in the low-affinity α_4_β_7_ with the bent conformation in the presence of Ca^2+^/Mg^2+^ and exhibited an eightfold increase in the high-affinity α_4_β_7_ with the extended conformation in the presence of Mn^2+^ (Fig. 1d). In the cells expressing other insertion mutants of β_7_, PA was exposed to be recognized by NZ-1 in the bent conformation, the same as in the extended conformation of β_7_ (Fig. 1d). These data showed that PAins2 in the PSI domain of β_7_ was an appropriate PA tag insertion design.

**Figure 2:**
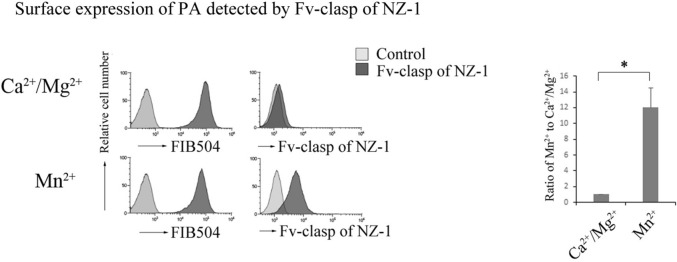
The Fv-clasp of NZ-1 recognized a conformational change in α_4_β_7_. (Left) Flow cytometry profiles of FIB504 or the Fv-clasp of NZ-1 of PAins2-expressing cells in the presence of 1 mM Ca^2+^/Mg^2+^, or 0.5 mM Mn^2+^. (Right) The binding of the Fv-clasp of NZ-1 to PAins2-expressing cells in the presence of 1 mM Ca^2+^/Mg^2+^, or 0.5 mM Mn^2+^. The IMF of binding of PAins2-expressing cells to the Fv-clasp of NZ-1 in the presence of 0.5 mM Mn^2+^ was normalized to the IMF of anti-β_7_ (FIB504) and is presented as the fold-increase relative to that in the presence of 1 mM Ca^2+^/Mg^2+^ values of 1. Data represent the mean ± SE of three independent experiments. *P < 0.003, versus in the presence of Ca^2+^/Mg^2+^.

As exogenous addition of antibodies that preferentially bind to the extended conformation can shift the equilibrium toward the high-affinity state of integrins, they often activate integrins from outside the cell. Therefore, we confirmed the conformational change in α_4_β_7_ in the presence of Mn^2+^ using the Fv-clasp of NZ-1. The Fv-clasp of NZ-1, an artificially designed small antibody fragment of 37 kDa, was used as a reporter of conformational change, as it did not affect the equilibrium between the high- and low-affinity states^19^. Using Fv-clasp of NZ-1, the surface expression of PA tag exhibited a 11-fold increase in the presence of Mn^2+^, compared to that in the presence of Ca^2+^/Mg^2+^ (Fig. 2), indicating that the surface expression of PA precisely reflects the active conformation of α_4_β_7_.

### Rap1 deficiency induced a conformational change in α_4_β_7_

**Figure 3:**
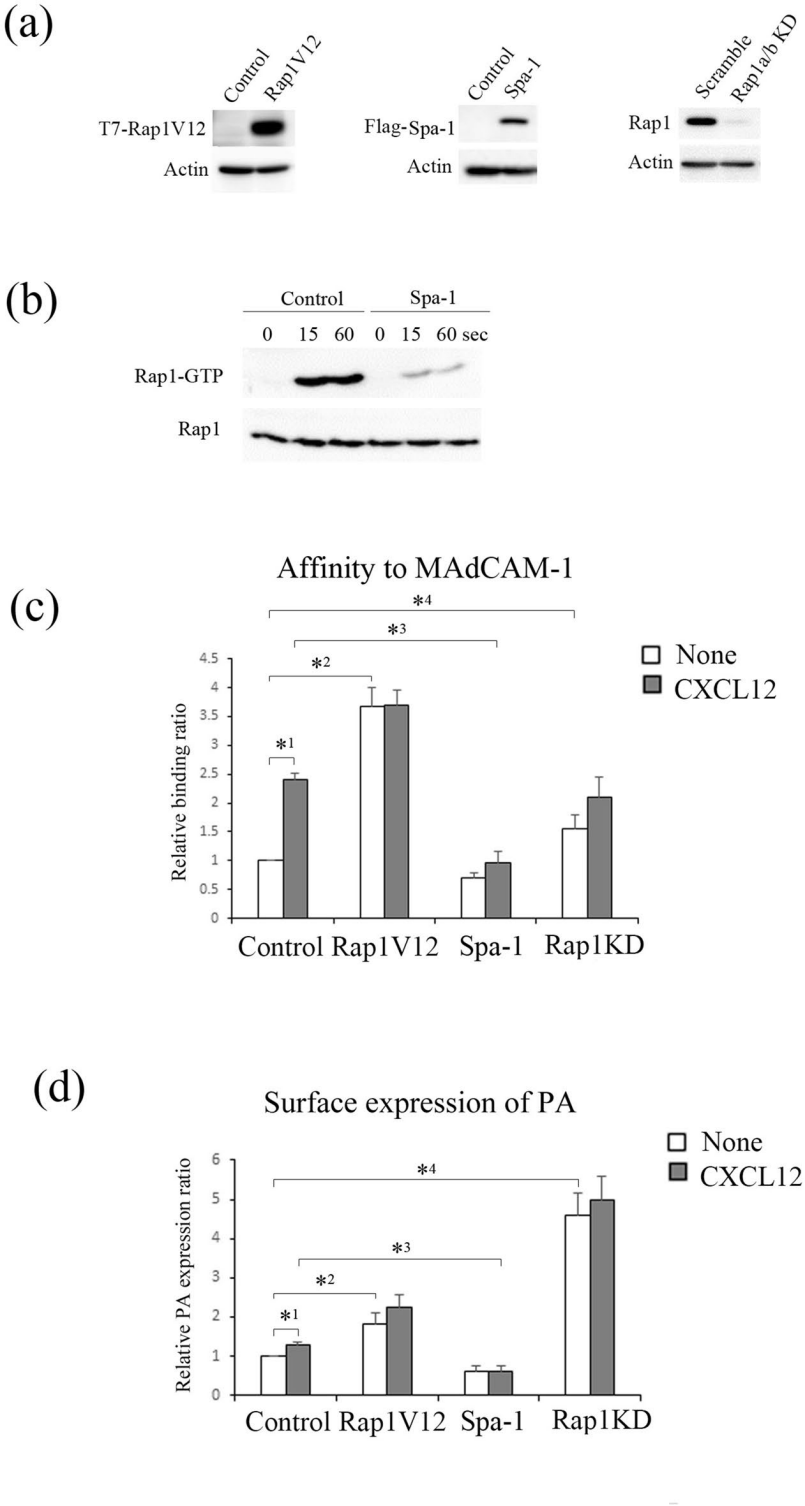
The conformation of α_4_β_7_ was regulated by Rap1. (**a**) Immunoblot of the total cell lysates from the cells introduced with control, T7-Rap1V12, flag-Spa-1-expressing, or *Rap1a/b*-knockdown cells with anti-T7, flag, or Rap1 antibodies. (**b**) GTP-bound Rap1 was analyzed by a pull-down assay using GST-Ral-GDS-RBD. Control or SPA-1-expressing cells were stimulated with 100 nM of CXCL12 at the indicated times, lysed, and subjected to a pull-down assay. Bound Rap1 and total Rap1 were detected by immunoblotting using an anti-Rap1 antibody. (**c**) Ligand binding affinity to soluble MAdCAM-1-Fc. The binding of each transfectant to soluble MAdCAM-1-Fc in the presence or absence of CXCL12. The IMF of soluble MAdCAM-1 binding was normalized to the IMF of anti-β_7_ (FIB504) and is presented as the fold-increase relative to that of unstimulated control cell values of 1. Data represent the mean ± SE of three independent experiments. *^1^*P* < 0.001, versus unstimulated cells, *^2^*P* < 0.001, versus unstimulated control cells, *^3^*P* < 0.006, versus CXCL12-stimulated control cells, *^4^*P* < 0.05, versus unstimulated control cells. (**d**) The binding of NZ-1 to each transfectant in the presence or absence of CXCL12. The IMF of NZ-1 binding was normalized to the IMF of anti-β_7_ and is presented as the fold-increase relative to that of unstimulated control cell values of 1. Data represent the mean ± SE of three independent experiments. *^1^*P* < 0.001, versus unstimulated control cells, *^2^*P* < 0.001, versus unstimulated control cells, *^3^*P* < 0.001, versus CXCL12-stimulated control cells, *^4^*P* < 0.001, versus unstimulated control cells.

A conformational equilibrium between bent (low-affinity) and extended (high-affinity) states of integrin was found to be regulated by inside-out signaling such as Rap1. Using PAins2 cells, we examined the effects of Rap1 on the conformational state of α_4_β_7_. To this end, we introduced the GTP-binding form of Rap1, *Rap1V12*, Rap1 GTPase activating protein (GAP), *Spa-1*, and knocked down of *Rap1a/b* in PAins2 cells (Fig. 3a). We confirmed that CXCL12-induced Rap1 activation was inhibited in Spa-1-expressing PAins2 cells (Fig. 3b).

Next, we examined the binding activity of α_4_β_7_ on each transfectant to soluble MAdCAM-1 in the presence or absence of CXCL12. As shown in Fig. 3c, CXCL12 stimulation elevated the binding of α_4_β_7_ on control cells to soluble MAdCAM-1, indicating that chemokine stimulation shifted the equilibrium of α_4_β_7_ toward high-affinity state. As expected, overexpression of Rap1V12 increased the binding of α_4_β_7_ to soluble MAdCAM-1, without CXCL12 stimulation (Fig. 3c). The inhibition of Rap1 activation by overexpression of Spa-1 suppressed CXCL12-dependent increase in the binding of α_4_β_7_ to MAdCAM-1 (Fig. 3c). Knockdown of *Rap1* also significantly increased the binding activity, but the effect was weak compared to that of Rap1V12-expressing cells (Fig. 3c). These data suggest that Rap1-GDP locks α_4_β_7_ in the low-affinity state and that Rap1-GTP further promotes an equilibrium toward high-affinity state of α_4_β_7_.

Subsequently, we examined changes in the surface expression of the PA tag in each transfectant. CXCL12 stimulation exhibited a 1.3-fold increase in PA surface expression, and overexpression of Spa-1 completely inhibited PA surface expression induced by CXCL12 stimulation (Fig. 3d), indicating that the conversion of Rap1-GDP to GTP is necessary for the surface expression of PA. Overexpression of Rap1V12 significantly promoted PA surface expression (Fig. 3d). PA surface expression was higher in Rap1-deficient cells as compared with that in Rap1V12-expressing cells (Fig. 3d), suggesting that the loss of Rap1-GDP induced a conformational change in α_4_β_7_ and that this change is different from the Rap1V12-induced conformation of α_4_β_7_. These results indicate that Rap1-GDP suppresses conformational changes to active form of α_4_β_7_.

In addition, talin is reported to bind Rap1-GTP and integrin, and trigger integrin activation^20^. Therefore, we examined the effect of the knockdown (KD) of talin. The abundance of talin protein in *talin KD* cells was reduced to 5% of control cells (Fig. S1a). As shown in Fig. S1b, the silencing of talin reduced basal surface expression of PA, whereas CXCL12 increased surface expression of PA in talin-KD cells at a same proportion as control cells. This result suggests that talin is a basic cytoskeletal component necessary for active conformation of α_4_β_7_, rather than a downstream effector molecule of chemokine-mediated signaling.

### Identification and characterization of rat mAbs to detect Rap1-dependent conformational changes in α_4_β_7_

**Figure 4:**
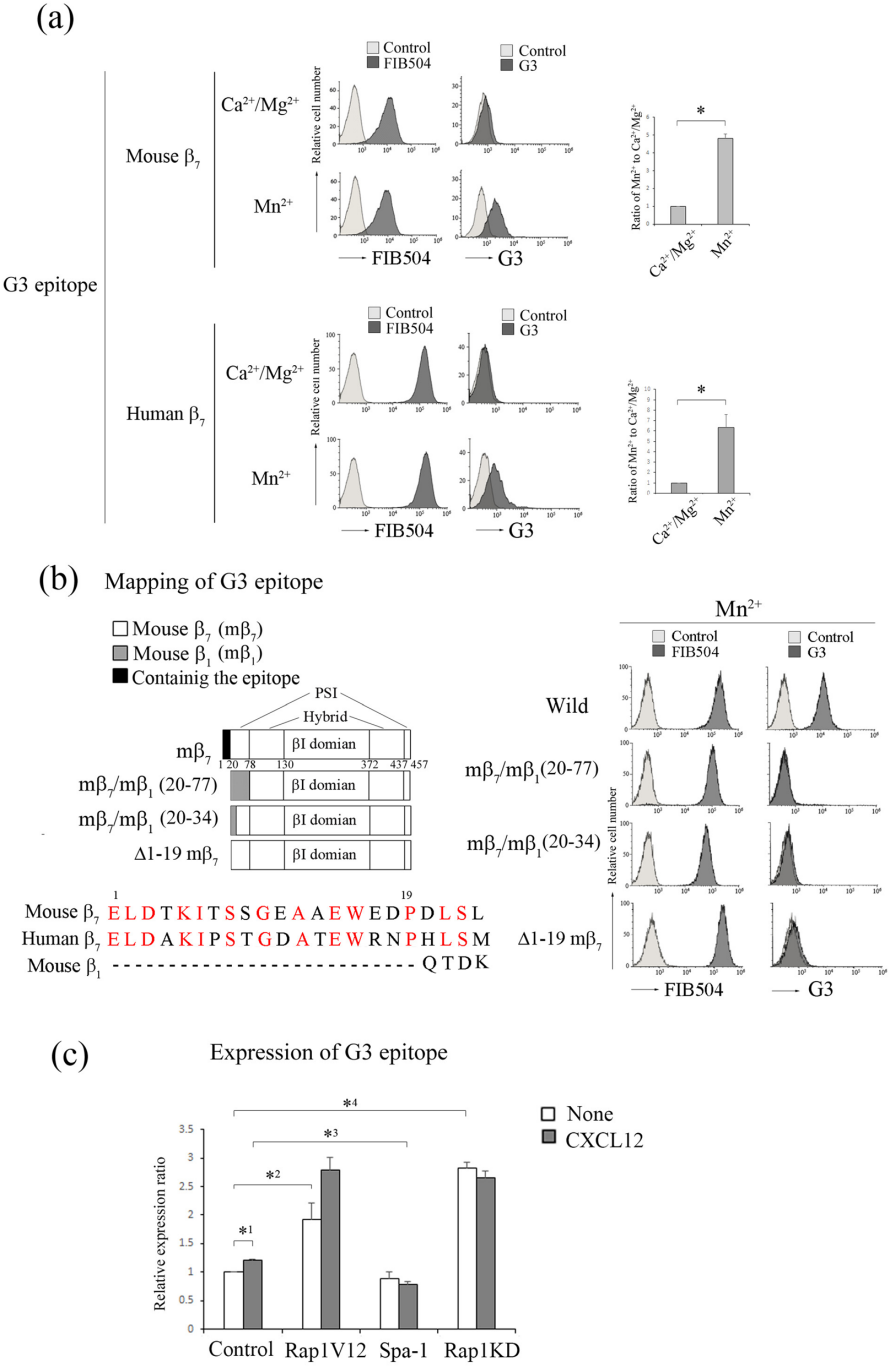
G3 mAb recognized the active conformation of α_4_β_7_. (**a**) (Upper) (left) Flow cytometry profiles of G3 mAb of wild-type (wt) BAF cells in the presence of 1 mM Ca^2+^/Mg^2+^, or 0.5 mM Mn^2+^. (right) The IMF of binding to G3 mAb was normalized to the IMF of anti-β_7_ (FIB504) and is presented as the fold-increase relative to that of wt BAF cells in the presence of 1 mM Ca^2+^/Mg^2+^ values of 1. Data represent the mean ± SE of three independent experiments. *P < 0.001, versus Ca^2+^/Mg^2^. (Lower) (left) Flow cytometry profiles of G3 of Jurkat cells expressing human β_7_ in the presence of 1 mM Ca^2+^/Mg^2+^, or 0.5 mM Mn^2+^. (right) The IMF of binding to G3 mAb was normalized to the IMF of anti-β_7_ and is presented as the fold-increase relative to that of Jurkat cells in the presence of 1 mM Ca^2+^/Mg^2+^ values of 1. Data represent the mean ± SE of three independent experiments. *P < 0.001, versus Ca^2+^/Mg^2+^. (**b**) (Left) (upper) Mapping of G3 epitope. G3 mAb reactivity was determined by co-expressing chimeric murine β_1_/β_7_ subunits or ∆1–19 murine β_7_ with endogenous α_4_ in β_7_-knockout BAF cells in the presence of 0.5 mM Mn^2+^ using flow cytometry. (lower) The amino acid sequence of 1–19 a.a. of murine, human β_7_ and murine β_1_. The red characters indicate the candidates recognized by G3 mAb. (right) Flow cytometry profiles of the transfectants with anti-β_7_ or G3 in the presence of 0.5 mM Mn^2+^. (**c**) The binding of G3 mAb to α_4_β_7_ on the cells introduced with control, Rap1V12, Spa-1-expressing, or *Rap1a/b*-knockdown cells in the presence or absence of CXCL12. The IMF of binding to G3 mAb was normalized to the IMF of anti-β_7_ and is presented as the fold-increase relative to that of unstimulated control cell values of 1. Data represent the mean ± SE of three independent experiments. *^1^*P* < 0.001, versus unstimulated cells, *^2^*P* < 0.03, versus unstimulated control cells, *^3^*P* < 0.002 versus CXCL12-stimulated control cells, *^4^*P* < 0.001, versus unstimulated control cells.

To establish hybridomas producing mAbs that exclusively reacted with α_4_β_7_ in an Mn^2+^-dependent manner, immunogenic N-terminal amino acids (1–458) of β_7_-MBP fusion protein were injected into rats. A hybridoma producing rat mAb G3 (γ2/κ) for Mn^2+^-dependent conformation of α_4_β_7_ was established. As shown in Fig. 4a, the G3 epitope was almost not detected in the low-affinity α_4_β_7_ with a bent conformation in the presence of Ca^2+^/Mg^2+^ but increased 4.8-fold in the high-affinity α_4_β_7_ with the extended conformation in the presence of Mn^2+^.

Next, we tested the cross-reactivity of the G3 mAb with human α_4_β_7_ using Jurkat cells transfected with human β_7_. The surface expression level of β_7_ was determined in the Jurkat cells using FIB504 (Fig. 4a). G3 epitope expression in the Jurkat cells was extremely low in the presence of Ca^2+^/Mg^2+^ and increased fivefold in the presence of Mn^2+^ (Fig. 4a). To identify the epitope of G3, we constructed murine β_1_/β_7_ chimeras and the deletion mutant (∆1–19) of β_7_. These mutants were co-expressed with endogenous α_4_ in β_7_-knockout BAF cells, and the surface expression level was confirmed by the immunostaining with FIB504 (Fig. 4b). As shown in Fig. 4b, the deletion of N-terminal 1–19 a.a. of β_7_, which does not exist in β_1_, let G3 mAb lose the reactivity to murine β_7_. Thus, flow cytometric analysis of these transfectants showed that the β_7_ segment 1–19 a.a. located in the PSI domain was required for binding of G3 mAb to β_7_ (Fig. 4b), which was close to the PA grafting site (Fig. 1c). As expected, G3 epitope expression increased 1.2-fold with CXCL12 stimulation (Fig. 4c). Overexpression of Rap1V12 enhanced the expression of G3 epitope to 1.9-fold (Fig. 4c). Rap1-deficiency also increased the expression of G3 epitope to 2.8-fold (Fig. 4c). Consistent with the results using the PA-tag-NZ-1 system, these data indicate that Rap1-GDP locks the conformation of α_4_β_7_ in inactive state.

**Figure 5:**
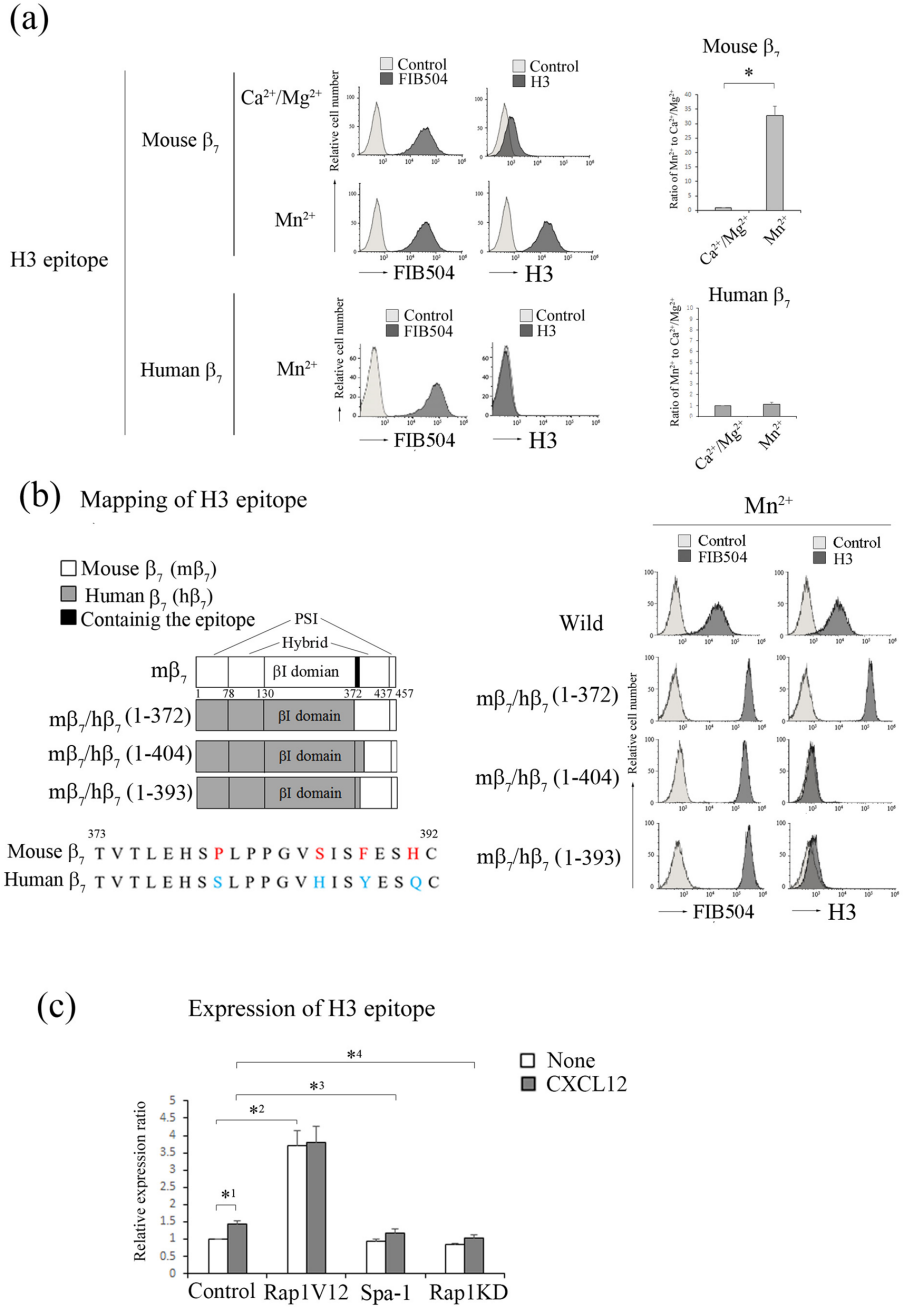
H3 mAb recognized the active conformation of α_4_β_7_. (**a**) (Upper) (left) Flow cytometry profiles of H3 mAb of wt BAF cells in the presence of 1 mM Ca^2+^/Mg^2+^, or 0.5 mM Mn^2+^. (right) The IMF of binding to H3 mAb was normalized to the IMF of anti-β_7_ (FIB504) and is presented as the fold-increase relative to that of wt BAF cells in the presence of 1 mM Ca^2+^/Mg^2+^ values of 1. Data represent the mean ± SE of three independent experiments. *P < 0.001, versus Ca^2+^/Mg^2+^. (Lower) (left) Flow cytometry profiles of H3 of Jurkat cells expressing human β_7_ in the presence of 0.5 mM Mn^2+^. (right) Normalized IMF of binding to H3 mAb is presented as the fold-increase relative to that of Jurkat cells in the presence of 1 mM Ca^2+^/Mg^2+^ values of 1. Data represent the mean ± SE of three independent experiments. (**b**) (Left) (upper) Mapping of H3 epitope. H3 mAb reactivity was determined by co-expressing chimeric murine/human β_7_ subunits with endogenous α_4_ in β_7_-knockout BAF cells in the presence of 0.5 mM Mn^2+^ using flow cytometry. (lower) The amino acid sequence of 373–393 a.a. of murine and human β_7_. The red characters indicate the candidates recognized by H3 mAb. The blue characters indicate the corresponding human amino acid sequence. (right) Flow cytometry profiles of the transfectants with anti-β_7_ or H3 mAb in the presence of 0.5 mM Mn^2+^. (**c**) The binding of H3 mAb to α_4_β_7_ on the cells introduced with control, Rap1V12, Spa-1-expressing, or *Rap1a/b*-knockdown cells in the presence or absence of CXCL12. The IMF of binding to H3 mAb was normalized to the IMF of anti-β_7_ and is presented as the fold-increase relative to that of unstimulated control cell values of 1. Data represent the mean ± SE of three independent experiments. *^1^*P* < 0.02, versus unstimulated cells, *^2^*P* < 0.005, versus unstimulated control cells, *^3^*P* < 0.05 versus CXCL12-stimulated control cells, *^4^*P* < 0.05 versus CXCL12-stimulated control cells.

We also established a hybridoma producing rat mAb H3 (γ2/κ) to detect Mn^2+^-dependent conformation of α_4_β_7_. As shown in Fig. 5a, the expression of H3 epitope was almost not detected in the low-affinity of α_4_β_7_ with the bent conformation in the presence of Ca^2+^/Mg^2+^ and increased 33-fold in the presence of Mn^2+^, suggesting that H3 mAb recognized the high-affinity α_4_β_7_. Subsequently, we explored the cross-reactivity of H3 mAb with human α_4_β_7_ using Jurkat cells transfected with human β_7_. H3 epitope on Jurkat cells was not expressed in the presence of Mn^2+^ (Fig. 5a), indicating that H3 mAb did not recognize the active conformation of human β_7_. To identify the epitope of H3, we constructed murine/human β_7_ chimeras. These chimeras were co-expressed with endogenous α_4_ in β_7_-knockout BAF cells, and the surface expression level was confirmed by immunostaining with FIB504 (Fig. 5b). Flow cytometric analysis of these transfectants showed that the β_7_ segment 373–393 a.a. located in the hybrid domain was required for binding of H3 mAb to β_7_ (Fig. 5b). As expected, the expression of H3 epitope also increased 1.4-fold with CXCL12 stimulation (Fig. 5c). Overexpression of Rap1V12 enhanced the expression of H3 epitope to 3.7-fold (Fig. 5c). However, Rap1 deficiency did not increase the expression of H3 epitope with or without CXCL12 stimulation (Fig. 5c). These data indicate that the expression of H3 epitope requires Rap1-GTP.

**Figure 6:**
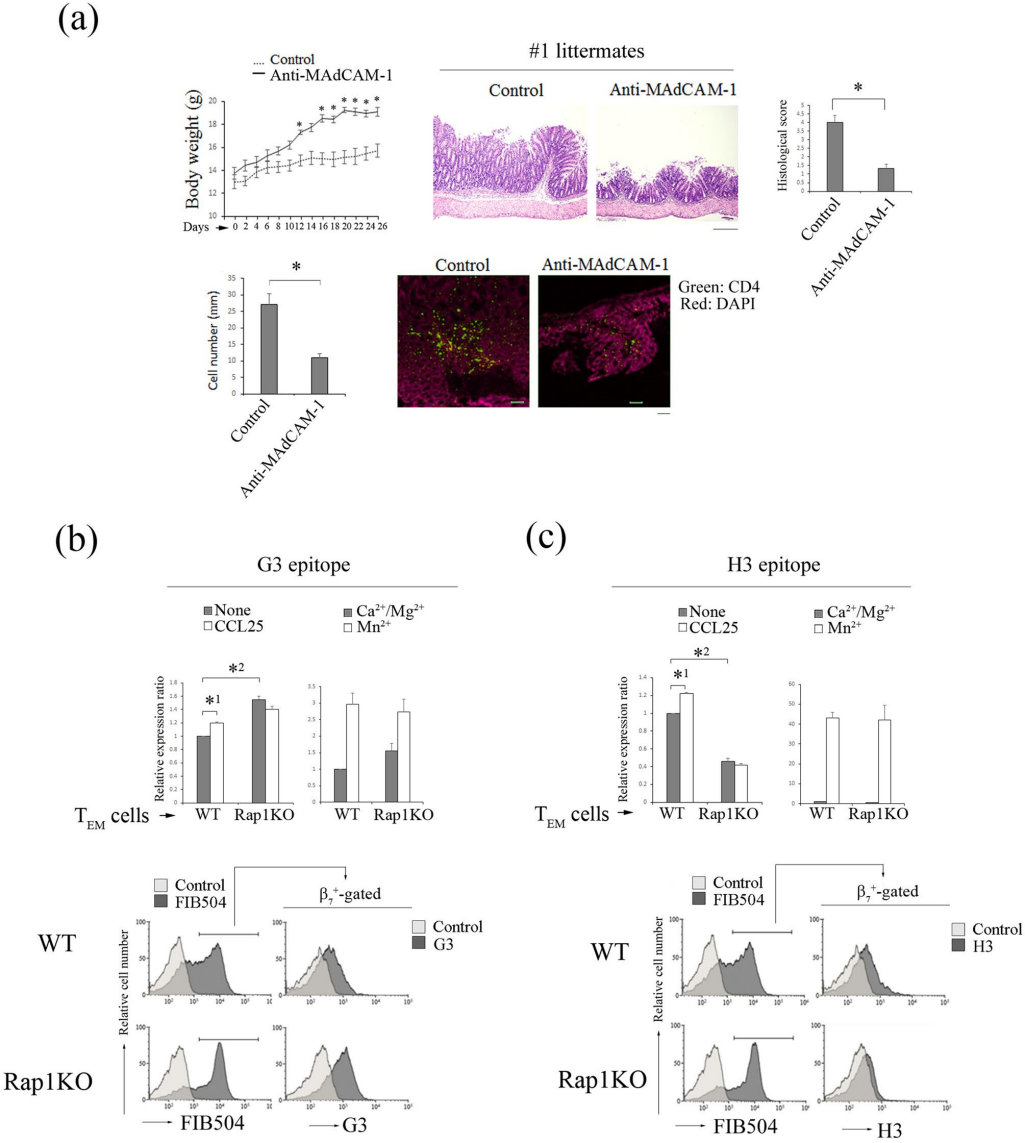
Expression of G3 epitope in colitis-causing CD4^+^ T_EM_ cells. (**a**) The administration of anti-MAdCAM-1 inhibited the development of spontaneous colitis in T cell-specific *Rap1a/b* knockout mice (colitis mice). (Upper) (left) The body weights of colitis mice injected with control or anti-MAdCAM-1 Ab are presented as percentages of the original body weight. (*n* = 3 in each group). *P < 0.05 versus control Ab. (middle) Histology of intestinal inflammation in a set (#1) of the littermates of colitis mice that received control or anti-MAdCAM-1 Ab (×40). Scale bar, 500 μm. (right) Light microscopic assessment of damages of colitis. (Lower) (left) The density of infiltrated CD4^+^ T cells in the colon of colitis mice was evaluated using an immunohistological study. *P < 0.02 versus control Ab. (right) Frozen sections of the colon from colitis mice that received control or anti-MAdCAM-1 Ab were stained with anti-CD4 (green) and DAPI (red). Low (×100) Scale bar, 100 μm. (**b**) The expression of G3 epitope in CD4^+^ T_EM_ cells. (Upper) The binding of G3 to wt and Rap1-deficient (Rap1KO) CD4^+^ (CD44^+^) T_EM_ cells in the presence or absence of CCL25 (left) and with 1 mM Ca^2+^/Mg^2+^, or 0.5 mM Mn^2+^ (right). The IMF of binding to G3 mAb was normalized to the IMF of anti-β_7_ (FIB504) and is presented as the fold-increase relative to that of unstimulated wt T cell values of 1. Data represent the mean ± SE of three independent experiments. *^1^*P* < 0.005, versus unstimulated wt cells. *^2^*P* < 0.001, versus unstimulated wt cells. (Lower) Flow cytometry profiles of anti-β_7_ on unstimulated wt and Rap1KO T_EM_ (CD44^+^CD62L^−^) cells, and G3 mAb on β_7_^+^-gated wt and Rap1KO T_EM_. (**c**) (Upper) The binding of H3 mAb to wt and Rap1 KO CD4^+^ (CD44^+^) T_EM_ cells in the presence or absence of CCL25 (left) and with 1 mM Ca^2+^/Mg^2+^, or 0.5 mM Mn^2+^ (right). The IMF of binding to H3 mAb was normalized to the IMF of anti-β_7_ and is presented as the fold-increase relative to that of unstimulated wt cell values of 1. Data represent the mean ± SE of three independent experiments. *^1^*P* < 0.001 versus unstimulated wt cells, *^2^*P* < 0.001 versus unstimulated wt cells. (Lower) Flow cytometry profiles of anti-β_7_ on unstimulated wt and Rap1KO T_EM_ (CD44^+^CD62L^-^) cells, and H3 mAb on β_7_^+^-gated wt and Rap1KO T_EM._

In our previous paper^10^, we demonstrated that T cell-specific Rap1-deficient mice developed severe colitis with infiltration of CD4^+^ T_EM_ cells into colon LP and that adoptive transfer of these cells into normal mice induced colitis. Previous study also demonstrated that α_4_β_7_-MAdCAM-1-dependent rolling was significantly promoted in Rap1-deficient CD4^+^ T_EM_ cells^10^. In the present study, the injection of a MAdCAM-1 inhibitory mAb into T cell-specific Rap1-deficient mice prevented the development of colitis (Fig. 6a, Fig. S3). These findings confirmed that the α_4_β_7_-MAdCAM-1 interaction was critical for the development of colitis in T cell-specific *Rap1a/b*-knockout mice. Therefore, we explored whether a conformational change in α_4_β_7_ was observed in pathogenic T cells. Since CCR9 and its ligand CCL25 are found to play essential roles in gut-homing of T_EM_ cells^21^, we used CCL25 for the stimulation of CD4^+^ T_EM_ cells. As shown in Fig. 6, G3 epitope significantly increased in pathogenic T cells as compared to that in wild-type T_EM_ cells, although the surface expression of α_4_β_7_ was elevated in the pathogenic T cells. CCL25 increased the expression of G3 epitope in control cells but not in pathogenic T cells (Fig. 6b). As expected, the expression of the epitope recognized by H3 mAb was reduced and not induced by CCL25 stimulation in pathogenic T cells, although the addition of Mn^2+^ induced H3 epitope in these cells at a similar level to that in wild-type T_EM_ cells (Fig. 6c). These data suggest that active conformation in α_4_β_7_ induced by Rap1 deficiency is sufficient for the infiltration of the pathogenic T cells into colon LP through rolling and arrest on MAdCAM-1-expressing endothelial cells.

In addition, previous study reports that CCL25 and CXCL10 induces different active conformation of α_4_β_7_^22^, but there is no difference between the effects of CCL25 and CXCL10 in the expression of G3 and H3 epitopes on CD4^+^ T_EM_ cells (Fig. S4).

## Discussion

**Figure 7:**
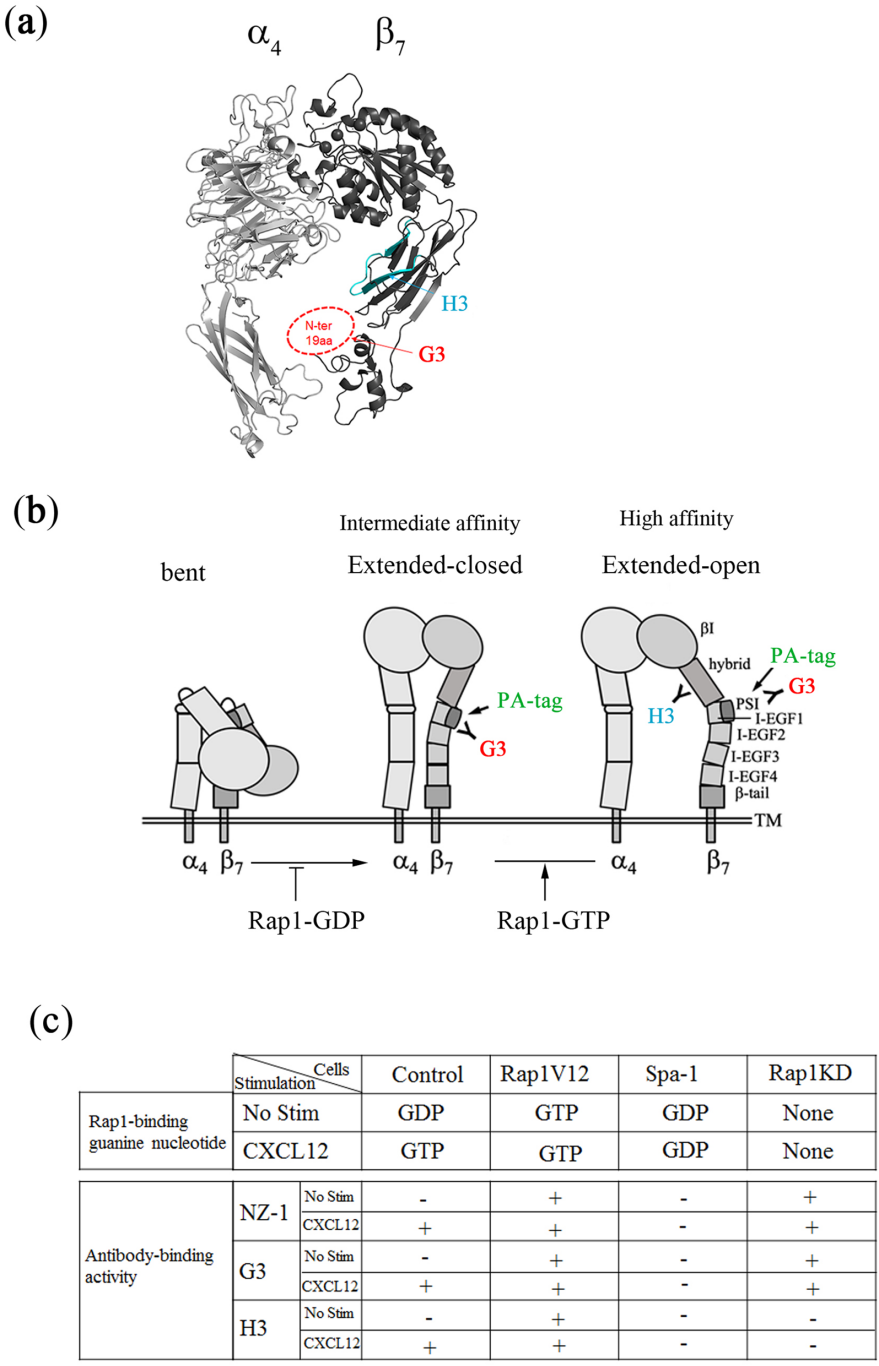
Model for the regulation of the conformation of α_4_β_7_ by Rap1. (**a**) The structure of the α_4_β_7_ headpiece. Crystal structure of α_4_β_7_ headpiece was derived from PDB database with PDB code of 3V4P^4^, and created using the PyMOL molecular visualization system. The PSI domain of the β_1_ headpiece structure is used instead of the PSI domain of β_7_, and the N-terminal 19 a.a. structure is unknown (surrounded by a dot-line). G3 mAb recognized the epitope in the PSI domain of β_7_ (red), and H3 mAb recognized the epitope in the hybrid domain of β_7_ (blue). (**b**) The model for conformational changes of α_4_β_7_. Both G3 (red) and the PA-tag located in PSI domain (green), and H3 (blue) epitopes were all hidden in the low affinity α_4_β_7_ with the bent conformation. The surface expression of G3 epitope and PA increased by Rap1 deficiency, which might have induced an “extended-closed form” of α_4_β_7_. Rap1-GDP restrained conformational changes in α_4_β_7_ and maintained the binding of α_4_β_7_ to MAdCAM-1 under conditions of low-affinity binding (the bent form of α_4_β_7_). The exposure of H3 epitope was dependent on Rap1-GTP, which was strongly correlated with the binding activity of α_4_β_7_ to soluble MAdCAM-1. Rap1-GTP may have induced swing-out of the hybrid domain, which corresponded to the “extended open form” of α_4_β_7_, resulting in the binding of α_4_β_7_ to MAdCAM-1 with high affinity. (**c**) Table summarizing the Rap1-binding guanine nucleotide (GTP or GDP) and each antibody (NZ-1, G3, H3)-binding activity (+ or −) in control, Rap1V12 or Spa-1-expressing cells, and Rap1KD cells in the presence or absence (No Stim) of CXCL12. The binding of the antibody to unstimulated control cells was displayed with (−), and the case that significantly increased, was displayed with (+).

In this study, we developed a sensitive system to probe the conformational states of α_4_β_7_ using the insertion of PA tag into the PSI domain of the β_7_ chain. Using this system, we found that the conformation of α_4_β_7_ was regulated by Rap1. To examine the conformational changes of β_7_ in primary lymphocytes, we isolated two rat mAbs (G3 and H3) against activation-dependent α_4_β_7_. G3 mAb recognized the epitope in the PSI domain of β_7_, and H3 mAb recognized the epitope in the hybrid domain of β_7_ (Fig. 7a). Both epitopes were hidden in the low-affinity α_4_β_7_ with the bent conformation and exposed in the high-affinity α_4_β_7_ with the extended conformation induced by the addition of Mn^2+^. The introduction of Rap1V12 induced the exposure of G3 and H3 epitopes to be recognized by mAbs. However, the expression of G3 epitope was increased by depletion of Rap1-GDP, whereas the conversion to Rap1-GTP was indispensable for the exposure of H3 epitope. Thus, Rap1-GDP and GTP independently regulated the conformation of α_4_β_7_ (Fig. 7).

The binding of NZ-1 or G3 mAb to the PSI domain of β_7_ was suppressed by Rap1-GDP, and loss of Rap1-GDP was sufficient for maximal expression of these epitopes (Fig. 7). On the other hand, H3 epitope in the hybrid domain of β_7_ was not exposed by only deletion of Rap-GDP. The loss of Rap1-GDP had marginal effect on the binding of α_4_β_7_ to soluble MAdCAM-1. The overexpression of Rap1V12 as well as the addition of Mn^2+^, which increased the binding of α_4_β_7_ to soluble MAdCAM-1, induced the exposure of H3 epitope (Figs. 3c, 5c). Thus, the surface expression of H3 epitope was strongly correlated with the binding activity of α_4_β_7_ to soluble MAdCAM-1. These findings indicate that H3 mAb might detect the swing-out of the hybrid domain in the β_7_ chain, which is predicted to stabilize the high-affinity conformation^23^ (Fig. 7). These data suggest that Rap1-GDP restrained the bent conformation in α_4_β_7_ and maintain the binding of α_4_β_7_ to MAdCAM-1 in low-affinity and that the conversion into Rap1-GTP further facilitated the active conformation of α_4_β_7_, resulting in binding of α_4_β_7_ to MAdCAM-1 in high-affinity (Fig. 7). As previously reported^24,25^, conformation-specific antibodies are useful for the elucidation of the functions and regulatory mechanisms of integrin conformation. The combination of G3 and H3 mAbs, which might differentiate extended closed from extended open conformation of α_4_β_7_, will contribute to various studies of conformational regulation of α_4_β_7_.

The insertion site of PA-tag was between the 29th and 30th in N-terminal of amino acid sequence and G3 epitope was in N-terminal first 19 amino acids. Previous study^26,27^ report that AP5 is a mAb that recognizes β3 integrin only in the extended conformation. These findings suggest that N-terminal epitope could be used in many or all beta integrins to obtain antibodies that recognize an active conformation. Since there are many kinds of antibodies including N-terminal amino acids of β chain^28^, it is necessary to clarify whether these antibodies specifically recognize the active conformation of other integrin.

In a previous study, we demonstrated that Rap1-GDP activated LOK and promoted ERM phosphorylation and that the introduction of the active form of LOK or phospho-mimetic ezrin did not prevent conformational changes in α_4_β_7_. Although RIAM (Rap1-interacting molecule) and talin are known to induce active conformation of integrins, they are associated with Rap1-GTP but not Rap1-GDP^8,9,29^. In this study, we suggest that talin is a basic cytoskeletal component involved in active conformation of α_4_β_7_. Studies are needed to shed light on what are the downstream effector molecules of Rap1-GDP/GTP and the roles of cytoskeletal proteins such as RIAM and talin in regulation of conformation of α_4_β_7_.

Previous paper^22^ demonstrates that CCL25 and CXCL10 induce different conformation of α_4_β_7_, which favors a MAdCAM-1- and VCAM-1-binding, respectively. According to that paper, CCL25 and CXCL10 activates the different signaling pathways which lead to different phosphorylation states of β_7_ and distinct talin and kindling-3 binding patterns^22^. On the other hand, there was no difference in surface expression of G3 and H3 epitopes between CCL25 and CXCL10-stimulated T cells (Fig. S4). Therefore, G3 and H3 mAbs did not seem to discriminate the MAdCAM-1 or VCAM-1-binding conformation of α_4_β_7_. In this study, the conformation of α_4_β_7_ recognized by G3 mAb was suggested to be critical for gut-homing of T_EM_ cells, whereas the physiological significance of Rap1-GTP-dependent active conformation recognized by H3 mAb remains to be solved_._ It is important to clarify the biological implication of conformational regulation.

Rap1 is indispensable for chemokine-dependent integrin activation and naive lymphocyte recirculation, and its deficiency leads to lymphopenia in secondary lymph nodes^10,30^. On the other hand, Rap1-deficient T_EM_ cells exhibit enhanced α_4_β_7_/MAdCAM-1-dependent rolling and arrest on the endothelium, as well as accelerated homing into colon LP^10^. In this study, we found that Rap1 deficiency in T_EM_ cells led to a conformational change in α_4_β_7_, which promoted α_4_β_7_/MAdCAM-1-dependent endothelial transmigration and homing to colon LP^10^. Furthermore, the Rap1-GTP-dependent high-affinity conformation of α_4_β_7_, which was recognized by H3 mAb, was unnecessary for homing of pathogenic T cells into colon LP.

G3 mAb recognized the murine and human active form of α_4_β_7_, and the expression of G3 epitope correlated with the infiltration of pathogenic T_EM_ cells into colon LP. Thus, this mAb might be a useful tool to deliver drugs to pathogenic T_EM_ cells. In addition, as the inhibition of the conformational change in α_4_β_7_ seemed to be effective in preventing the infiltration of T_EM_ cells into colon LP, the system we developed can be used to screen for drugs to treat colitis. By recognizing conformational changes in α_4_β_7_, the system can serve as a useful tool for studies on α_4_β_7_ activation mechanisms and the development of new therapies for colitis and subsequent colorectal cancer.

## Methods

### Mice

All animal experiments were carried out in accordance with Regulations for the Care and Use of Laboratory Animals in Kitasato University, and the protocols used in this study were ethically approved by the Institutional Animal Care and Use Committee for Kitasato University.

*Rap*1a^f/f^ mice containing floxed exons 2–3 of *Rap1a*, *Rap1b*^f/f^ mice containing floxed exon 1 of *Rap1b* were maintained under specific pathogen–free conditions. Those mice were crossed with CD4-Cre mice, yielding mice with T cell-specific deletion of *Rap1a/b*^10^.

### Cell lines

Ba/F3 cells (BAF cells) and Jurkat cells were cultured as previously reported^31^. BAF cells were cultured with RPMI1640 medium containing 10% fetal calf serum, 50 mM beta-mercaptoethanol, and 1% WEHI-3 conditional medium as a source of interleukin 3. Jurkat cells were maintained with RPMI1640 medium containing 10% fetal calf serum. All cell lines were tested for mycoplasma contamination by 4′,6-diamidino-2-phenylindole (DAPI) staining with negative results.

### Antibodies and reagents

Fluorescence-conjugated anti-mouse CD4, CD44, anti-β_7_ (FIB504) (BioLegend), anti-Rap1(BD Biosciences), β-actin (Sigma), T7 (MBL) , Flag (Wako), anti-talin (Abcam), APC-conjugated anti-Rat or human IgG, and peroxidase-conjugated goat anti-Rabbit or -mouse IgG (Cell Signaling) were used for flow cytometry and immunoblotting. Anti-MAdCAM-1(MECA-367) (ATCC), G3 and H3 mAb were purified using HiTrap Protein G HP (GE healthcare). Mouse CXCL12, CCL25 and CXCL10 were purchased from R&D Systems. The single-chain Fv of NZ-1 (Fv-clasp) was created by fusing an anti-parallel coiled-coil structure derived from the SARAH domain of human Mst1 kinase to the fragments of V_H_ and V_L_ of NZ-1. NZ-1V_H_-SARAH and V_L_-SARAH were separately expressed in *E. coli* strain BL21, and cultured in standard LB media^19^. After the cell lysis by sonication, the denatured and solubilized V_H_-SARAH and V_L_-SARAH chains were then mixed, and the denaturing reagent was diluted to promote protein folding, and correctly-folded, disulfide-bonded Fv-clasp was purified as previously reported^19^. Fv-clasp was fluorescently labeled with Alexa Fluor 647 Amine-Reactive Dye (Thermo Fisher Scientific).

### DNA constructs and transfection

cDNA encoding murine *β*_*7*_ cDNA was subcloned into a pcDNA3.1 vector. Then, *β*_7_ mutants with a PA tag were generated from a pcDNA3.1-murine *β*_7_ construct using inverse PCR. The following oligonucleotides and their corresponding complimentary strands were used: for PAins 1: 5′-CCTGACCTGTCTCTGCAGGGCGTTGCCATGCCAGGTGCCGAAGATGATGTGGTGGGATCCTGCCAGCCAGTT-3′; for PAins 2: 5′-GGATCCTGCCAGCCAGTTGGCGTTGCCATGCCAGGTGCCGAAGATGATGTGGTGCCTTCCTGCCAGAAGTGT-3′: for PAins 3: 5′-TGGGTCACTCTTCAAGCTACTGGCGTTGCCATGCCAGGTGCCGAAGATGATGTGGTGCACTGCCTCCCAGAAGCCCAC-3′: and for PAins 4: 5′-CAAGCTACTCACTGCCTCCCAGGCGTTGCCATGCCAGGTGCCGAAGATGATGTGGTGGAAGCCCACGTCCTACGA-3′. The insertion positions are shown in Fig. 1c. To generate expression constructs of the PAins mutants, they were subcloned into an *EcoRI/XbaI* site of a lentivirus vector (CSII-EF-MCS; a gift from H. Miyoshi, RIKEN, Wako, Japan). A *Rap1V12* mutant and *Spa-1* were generated as previously described^32^. The fidelity of all the constructs was verified by sequencing.

### Generation of a hybridoma producing mAbs G3 or H3

DNA encoding N-terminal 1–458 amino acids of murine β_7_ was subcloned into a pMALc2x vector and that vector was transformed into *E.coli* BL21 competent cells. To synthesize recombinant maltose binding protein (MBP)-β_7_, BL21 were cultured at 37 °C to reach an OD600 of 0.4–0.6, and then isopropyl β-D-thiogalactopyranoside was added to a concentration of 0.2 mM and incubated at 30 °C for 3 h. The cells were lysed in lysis buffer (0.1% Triton X-100, 20 mM Tris–HCl, 200 mM NaCl, and 1 mM EDTA). MBP-β_7_ was purified from cell lysates using a pMAL Protein Fusion and Purification System (New England Biolabs).

WKY rats (8 week old) were injected intramuscularly at the tail base with an antigen emulsion containing MBP-β_7_ and Freund’s complete adjuvant (BD Biosciences). Then, 2 weeks later, lymphocytes were collected from iliac lymph nodes and fused with a murine myeloma cell line, SP2/0, using GenomeONE (ISHIHARA SANGYO)^33^. Hybridoma clones producing mAbs against the active form of β_7_ were screened by flow cytometry of BAF cells using the hybridoma supernatant in the presence or absence of Mn^2+^.

### RNA-mediated interference and gene introduction via lentiviral transduction

RNA-mediated interference was used to suppress mouse expression. As previously reported^34^, a 19-nucleotide -specific sense RNA sequences or a scrambled control RNA sequence of (Rap1a: 5′-GAATGGCCAAGGGTTTGCA-3′, Rap1b: 5′-AGACACTGATGATGTTCCA-3′, and talin: 5′-CGGTGAAGACTATCATGGT-3′) were introduced into BAF cells using a lentivirus vector with GFP (a gift from Dr. Miyoshi H., RIKEN, Wako, Japan) containing the RNAi construct under control of the H1 promoter cassette, respectively. The production and concentration of lentivirus particles were assessed as previously described^35^. The transduction efficiencies were greater than 90%. A GFP high population was collected by cell sorting and subjected to adhesion assays and immunoblot analysis.

### Immunoblot analysis

BAF cells were lysed in buffer (1% Nonidet P-40, 150 mM NaCl, 25 mM Tris–HCl [pH 7.4], 10% glycerol, 2 mM MgCl_2_, 1 mM phenylmethylsulfonylfluoride, 1 mM leupeptin, and 0.1 mM aprotinin). Cell lysates were subjected to immunoblotting^32^.

### Pull-down assays

Rap1-GTP was pulled down with a glutathione *S*-transferase (GST)-RBD of RalGDS fusion protein, respectively^36^. Briefly, 10^7^ cells were lysed in ice-cold lysis buffer (1% Triton X-100, 50 mM Tris–HCl [pH 7.5], 100 mM NaCl, 10 mM MgCl_2_, 1 mM phenylmethylsulfonyl fluoride, 1 mM leupeptin, and 0.5 mM aprotinin) and incubated for 1 h at 4 °C with GST-fusion proteins coupled to glutathione agarose beads. The beads were washed three times with lysis buffer and subjected to immunoblot analysis using an anti-Rap1 antibody. Immunoblotting of total cell lysates (5 × 10^4^ cells) was also performed.

### Assessment of activation epitopes by mAbs staining

Immunofluorescence flow cytometry was performed as described previously^31^. For NZ-1, G3 or H3 mAbs staining, cells were washed with binding buffer (0.1% BSA, 1 mM CaCl_2_, 1 mM MgCl_2_ or 0.1% BSA, 0.5 mM MnCl_2_ in HBSS), resuspended in 50 μl of the same buffer, and incubated for 30 min at 37 °C with 10 μg/ml of each mAb in the presence or absence of 0.5 μM CXCL12, CCL25 or CXCL10. Mean fluorescence intensities were measured using a Gallios flow cytometry or CytoFLEX (Beckman Coulter).

### Generation of β_7_-deficient BAF cells by the CRISPR/Cas9 system

The guide sequence targeting exon of the mouse β_7_ was cloned into pX330 (Addgene #42230)^37^. pX330-U6-Chimeric_BB-CBh-hSpCas9 was a gift from Feng Zhang (Addgene plasmid #42230, https://n2t.net/addgene:42230; RRID: Addgene_42230). pCAG-EGxxFP was used to examine efficiency of the target DNA cleavage by the guide sequence and Cas9 activity. The resultant guide sequence was cloned into GFP expressing plasmid DNA pX458 (Addgene #48138)^38^. pSpCas9(BB)-2A-GFP (PX458) was a gift from Feng Zhang (Addgene plasmid #48138, https://n2t.net/addgene:48138; RRID: Addgene_48138). The pX458 plasmid was transfected into BAF cells. 24 h after transfection, cells were sorted GFP-high population, followed by limiting dilution. Expression of full length β_7_ protein in each isolated clone was tested by flowcytometry. The sequence of the primer used were as follows: β_7_ Exon, Forward: 5′-GGGTCGACGCTGTGGAGTGAGTGAACTG-3′ and Reverse: 5′-GGGAATTCCTCTGAAGCCCAGTGCATTC-3′. Exon of guide sequence: Forward: 5′-CACCCACCTGGTCGCAGCGTGACG-3′ and Reverse: 5′-AAACCGTCACGCTGCGACCAGGTG-3′. Exon of β_7_ from edited clones was PCR amplified and verified by sequencing.

### Epitope mapping of G3 and H3

Human/murine β_7_, murine β_1_/β_7_ chimeras or ∆1–19 murine β_7_ were constructed using an In-Fusion HD cloning kit (TaKaRa). The In-Fusion HD enzyme premix fuses multiple PCR-generated sequences and linearized vectors efficiently and precisely, utilizing a 20-bp overlap at their ends. This 20-bp-overlap allows complementary base pairs between two pieces of DNA to anneal, leading to fragment joining. Therefore, when individual DNA fragments derived from human β_7_, murine β_1_ or β_7_ were amplified by PCR, a 20-bp overlap was engineered by designing custom primers (Table S1). The objective DNA fragments with a 20-bp overlap were joined into a linearized CSII-EF-MCS-IRES2-venus vector. The constructs were transduced to β_7_-knockout BAF cells by lentivirus. The binding of G3 or H3 to the BAF cells expressing the chimera β_7_ was measured in the presence of 0.5 mM Mn^2+^ by flow cytometry.

### Anti-MAdCAM-1 antibody treatment of colitis

T cell-specific *Rap1a/b* knockout mice aged 4 or 5 weeks were injected intraperitoneally with PBS containing 1 mg of rat IgG or anti-MAdCAM-1 antibody^39^ on days 0, 7, and 21. Their body weights were measured every 2 days. Pathological or frozen sections were prepared on day 28.

### Histological examination

Colon sections were fixed in 10% buffered formalin and embedded in paraffin. Paraffin-embedded colon sections were cut (3 μm), stained with haematoxylin and eosin and examined on an Olympus IX51 light microscope equipped with a CCD (charge-coupled device) camera. Tissues were graded semiquantitatively as described before^10,40^. Histological grades were assigned in a blinded manner on a scale of 0–5, as follows: grade 0, no changes observed,grade 1, minimal scattered mucosal inflammatory cell infiltrates, with or without minimal epithelial hyperplasia,grade 2, mild scattered to diffuse inflammatory cell infiltrates, sometimes extending into the submucosa and associated with erosions, with mild to moderate epithelial hyperplasia and mild to moderate mucin depletion from goblet cells; grade 3, moderate inflammatory cell infiltrates that were sometimes transmural, with moderate to severe epithelial hyperplasia and mucin depletion; grade 4, marked inflammatory cell infiltrates that were often transmural and associated with crypt abscesses or occasional ulceration, with marked epithelial hyperplasia, mucin depletion; and grade 5, marked transmural inflammation with severe ulceration or loss of intestinal glands.

### Immunostaining

Preparation of frozen sections of the colon from colitis mice were performed as described previously. Sections were blocked for 1 h at 20 °C with PBS containing 10% goat serum and 0.1% Triton X-100 and incubated overnight at 4 °C with APC conjugated anti-CD4 antibody. Nuclei were stained with SlowFade Gold antifade reagent with DAPI (invitrogen). Sections were examined on TCS SP8 (Leica).

### Measurement of soluble MAdCAM-1 binding.

The binding of mouse MAdCAM-1-Fc to BAF cells was measured as described before^10^. Cells were suspended in 50 μl binding buffer (0.1% BSA, 1 mM CaCl_2_, 1 mM MgCl_2_ or 0.1% BSA, 0.5 mM MnCl_2_ in HBSS), and 2 × 10^5^ cells/50 μl were then incubated with mouse MAdCAM-1-Fc (30 μg/ml)^34^. After two washes, samples were incubated for 20 min on ice with APC-conjugated mouse antibody to human IgG Fc-specific antibody (1 μg/ml). Unbound secondary antibody was removed by washing. Mean fluorescence intensities were measured using Gallios flow cytometry (Beckman Coulter).

### Statistical analysis

Statistical analysis was performed using two-tailed Student’s t-test. *P* values less than 0.05 were considered significant.

## Supplementary information

Supplementary information.
