# Supplementary material for: Dissection of α4β7 integrin regulation by Rap1 using novel conformation-specific monoclonal anti-β7 antibodies
Source: Sci Rep. 2020 Aug 6;10:13221. doi: 10.1038/s41598-020-70111-0 (PMC7413538; doi:10.1038/s41598-020-70111-0)
Supplement: Supplementary file 1 — Supplementary information. [file 41598_2020_70111_MOESM1_ESM.pdf]

Supplementary Information

Dissection of $\alpha_4\beta_7$ integrin regulation by Rap1 using novel conformation-specific monoclonal anti-β_7 antibodies.

Tsuyoshi Sato, Sayaka Ishihara, Ryoya Marui, Junichi Takagi and Koko Katagiri.

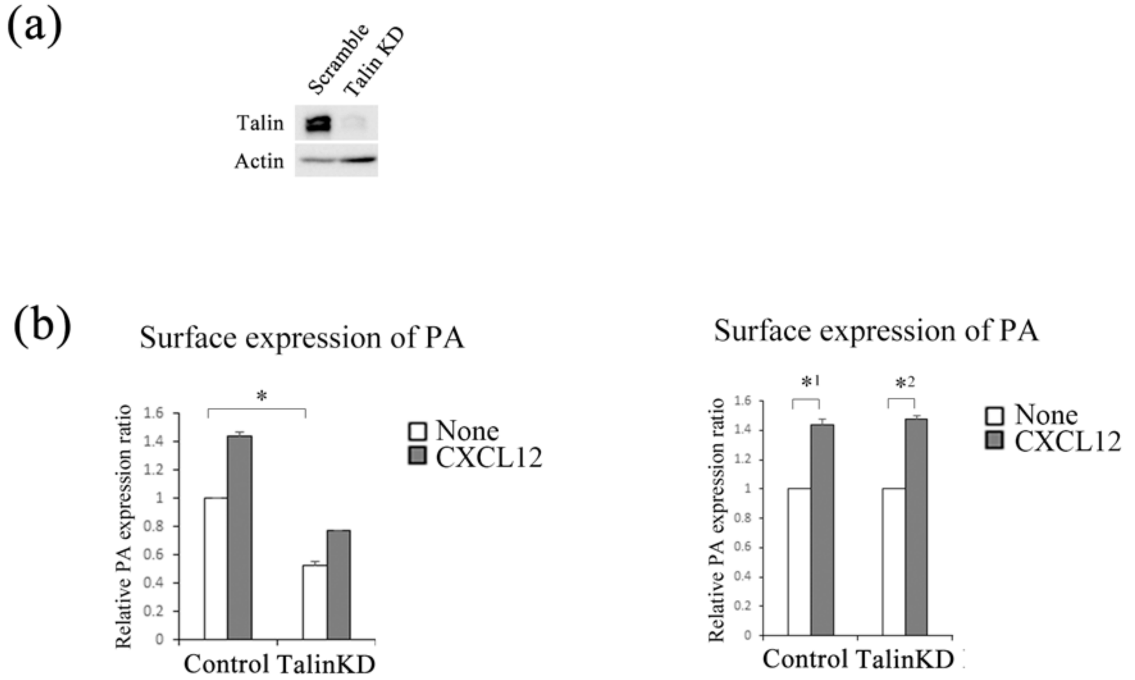

Figure S1. Effects of talin knockdown on surface expression of PA.

(a) Immunoblot of the total cell lysates from control and *talin*-knockdown (KD) cells with anti-talin antibody.

(b) (Left) The binding of NZ-1 to control and talin KD cells in the pesence or absence of CXCL12. The IMF of NZ-1 binding was normalized to the IMF of FIB504 and is presented as the fold-increase relative to that of unstimulated control cell values of 1. Data represent the mean ±SE of three independent experiments. **P* <0.001, versus unstimulated control cells.

(Right) The IMF of NZ-1 binding was normalized to the IMF of FIB504 and is presented as the fold-increase relative to that of each unstimulated control or *talin*-KD cell values of 1. *[1]*P* <0.001, versus unstimulated control cells. *[2]*P* <0.001, versus unstimulated talin KD cells.

Fig. 3a

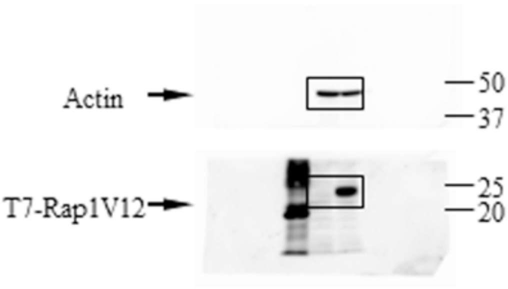

Fig. 3a

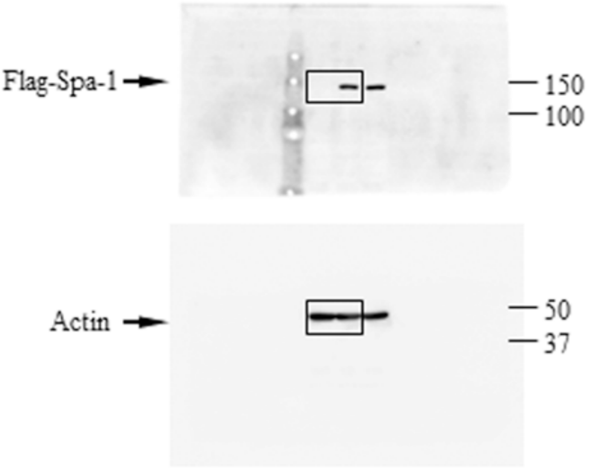

Fig. 3a

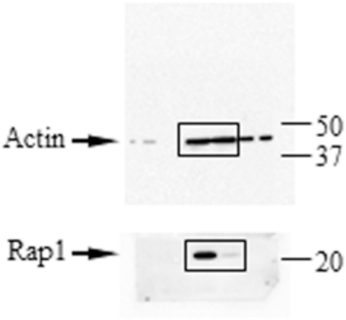

Fig. 3b

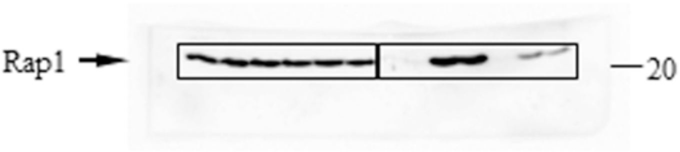

Fig. S1a

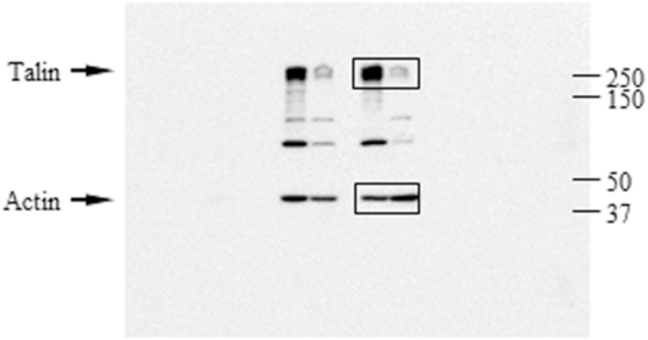

Figure S2. Full blots of immunoblotting shown in Figure 3a and 3b, and Figure S1a.

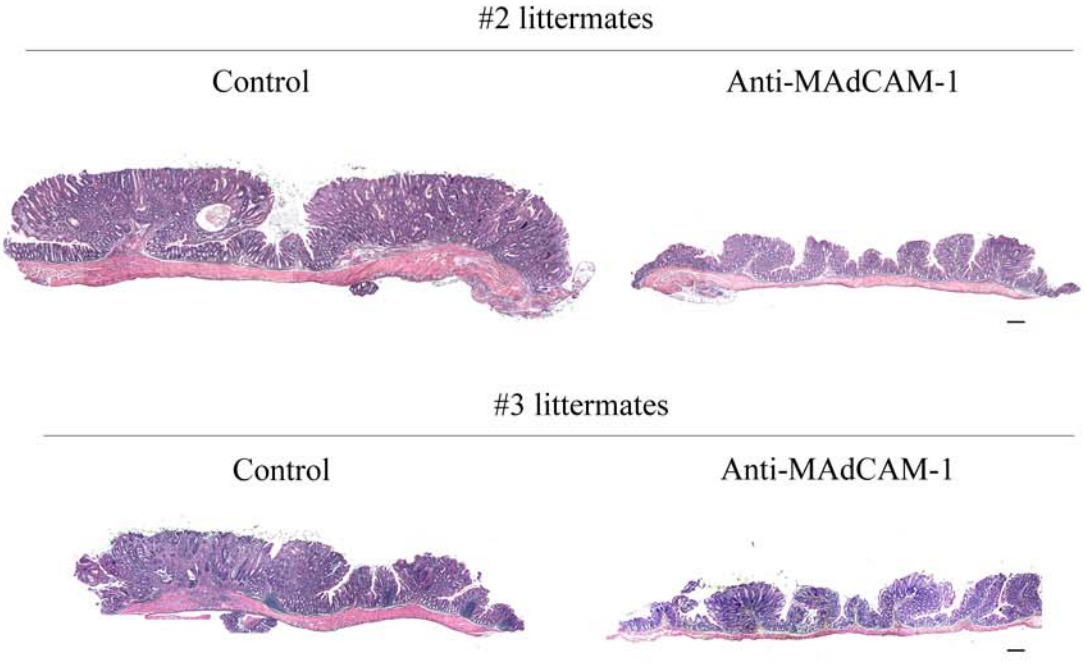

Figure S3. Histology of the colon derived from two sets (#2, #3) of two littermates which were injected with control antibody (Rat IgG) or anti-MAdCAM-1 antibody, respectively (x 40). Scale bar, 500 μm.

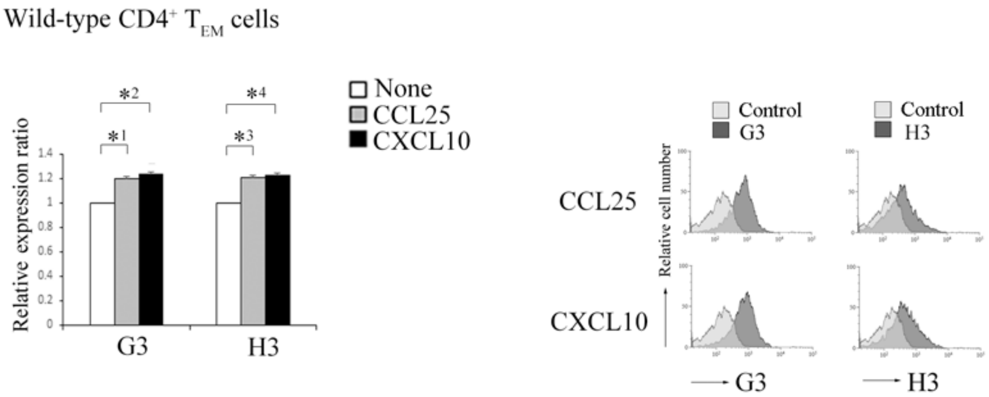

Figure S4. Surface expression of G3 or H3 epitope in $CD4^+$ T_{EM} cells upon CCL25 or CXCL10.

(Left) The surface expression of G3 or H3 epitope in wild-type (wt) $CD4^+$ T_{EM} cells.
The binding of G3 to wt $CD4^+$ ($CD44^+$) T_{EM} cells in the presence or absence of CCL25 or CXCL10.
The IMF of binding to G3 or H3 mAbs was normalized to the IMF of FIB504
and is presented as the fold-increase relative to that of unstimulated wt T_{EM} cells values of 1.
Data represent the mean±SE of three independent experiments. $*^1P < 0.005$, $*^2P < 0.001$, $*^3P < 0.001$, $*^4P < 0.001$, versus unstimulated cells.

(Right) Flow cytometry profiles of G3 or H3 on β_7^+-gated wt T_{EM} cells upon stimulation with CCL25 or CXCL10.

Table S1. Primers used for PCR

mβ_7/ mβ_1 (20-77)	
mβ_1 (20-77) -s	5’-TCGAGAATTCTGCAGCGGCCGCGCCACCATGGACAGCAAAGGTTCGTC-3’
mβ_1 (20-77) -as	5’-TCGGCTCCTCTATGTCACTTGGCTGGCAAC-3’
mβ_7 (78-787) -s	5’-AAGTGACATAGAGGAGCCGAGAGGCCGCCA-3’
mβ_7 (78-787) -as	5’-AGGGGCGGATCCGCGGCCGCTCAGTCTGCTTCCCTGGTCA-3’
mβ_7/ mβ_1 (20-34)	
mβ_1 (20-34) -s	5’-TCGAGAATTCTGCAGCGGCCGCGCCACCATGGACAGCAAAGGTTCGTC-3’
mβ_1 (20-34) -as	5’-AGAGAATACATTCTCCGCAAGATTTGGCAT-3’
mβ_7 (35-787) -s	5’-TTGCGGAGAATGTATTCTCTCACATCCCAG-3’
mβ_7 (35-787) -as	5’-AGGGGCGGATCCGCGGCCGCTCAGTCTGCTTCCCTGGTCA-3’
Δ1-19 mβ_7	
signal-s	5’-TCGAGAATTCTGCAGCGGCCGCGCCACCATGGACAGCAAAGGTTCGTC-3’
signal -as	5’-TCCCTGCAGAGACAGGGATCCGTCGACATCATCAT-3’
mβ_7 (20-787) -s	5’-GATCCCTGTCTCTGCAGGGATCCTGCCAG-3’
mβ_7 (20-787) -as	5’-AGGGGCGGATCCGCGGCCGCTCAGTCTGCTTCCCTGGTCA-3’
mβ_7/ hβ_7 (1-372)	
hβ_7 (1-372) -s	5’-TCGAGAATTCTGCAGCGGCCGCGCCACCATGGACAGCAAAGGTTCGTC-3’
hβ_7 (1-372) -as	5’-CAAGAGTCACAGTGGAAGACAGGCTATTATAAGCATCC-3’
mβ_7 (373-787) -s	5’-ATAGCCTGTCTTCCACTGTGACTCTTGAGCACTC-3’
mβ_7 (373-787) -as	5’-AGGGGCGGATCCGCGGCCGCTCAGTCTGCTTCCCTGGTCA-3’
mβ_7/ hβ_7 (1-404)	
hβ_7 (1-404) -s	5’-TCGAGAATTCTGCAGCGGCCGCGCCACCATGGACAGCAAAGGTTCGTC-3’
hβ_7 (1-404) -as	5’-GTCCCCGGTCCTCAGCCTTACCCTCCCTCTTCTC-3’
mβ_7 (405-787) -s	5’-TAAGGCTGAGGACCGGGGACAGTGCAATGATGTCC-3’
mβ_7 (405-787) -as	5’-AGGGGCGGATCCGCGGCCGCTCAGTCTGCTTCCCTGGTCA-3’
mβ_7/ hβ_7 (1-393)	
hβ_7 (1-393) -s	5’-TCGAGAATTCTGCAGCGGCCGCGCCACCATGGACAGCAAAGGTTCGTC-3’
hβ_7 (1-393) -as	5’-CAGGACCCTTACACTGGGATTCGTAAGAAATGTG-3’
mβ_7 (394-787) -s	5’-ATCCCAGTGTAAGGGTCCTGAGAAGACGGA-3’
mβ_7 (394-787) -as	5’-AGGGGCGGATCCGCGGCCGCTCAGTCTGCTTCCCTGGTCA-3’
